# The Challenge of Modulating Heparan Sulfate Turnover by Multitarget Heparin Derivatives

**DOI:** 10.3390/molecules25020390

**Published:** 2020-01-17

**Authors:** Noemi Veraldi, Nawel Zouggari, Ariane de Agostini

**Affiliations:** 1 Division of Clinical Pathology, Department of Diagnostics, Geneva University Hospitals, 1211 Geneva 14, Switzerland; noemi.veraldi@gmail.com; 2 Department of Biochemistry, Faculty of Science and Department of Pathology and Immunology, School of Medicine, University of Geneva, 1211 Geneva 14, Switzerland; nawel.zouggari@unige.ch; 3 Department of Pathology and Immunology, School of Medicine, University of Geneva and Division of Clinical Pathology, Department of Diagnostics, Geneva University Hospitals, 1211 Geneva 14, Switzerland

**Keywords:** heparan sulfate, heparin derivatives, neurodegenerative disorders, heparanase, cancer, mucopolysaccharidosis

## Abstract

This review comes as a part of the special issue “Emerging frontiers in GAGs and mimetics”. Our interest is in the manipulation of heparan sulfate (HS) turnover by employing HS mimetics/heparin derivatives that exert pleiotropic effects and are interesting for interfering at multiple levels with pathways in which HS is implicated. Due to the important role of heparanase in HS post-biosynthetic modification and catabolism, we focus on the possibility to target heparanase, at both extracellular and intracellular levels, a strategy that can be applied to many conditions, from inflammation to cancer and neurodegeneration.

## 1. Introduction

### 1.1. HS Turnover and the Role of Heparanase

Heparan sulfate (HS) is an anionic polysaccharide belonging to the glycosaminoglycan family that assembles as disaccharide building blocks of glucuronic acid (GlcA) linked α1-4 to N-acetyl-glucosamine (GlcNAc) and undergoes extensive modification through the action of at least four families of sulfotransferases and one epimerase. The sulfation of HS is carefully regulated in the ER-Golgi pathway where the first modification is introduced by N-deacetylase/N-sulfotransferase (NDST) enzymes and plays a key role in determining the subsequent modifications, dictating the position of the sulfated domains. Epimerization of some GlcA residues into iduronic acid (IdoA) can occur and it is followed by 2-O-sulfation of some iduronic acid residues, catalyzed by heparan sulfate 2-O- sulfotransferase (HS2ST), which can also act on some GlcA units. Next, on selected glucosamine residues sulfate groups are introduced by HS6ST1-3 transferases in the 6-O position and, finally, the relatively rare 3-O-sulfation is catalyzed by HS3ST1-6 [1]. Not only the length, but also the pattern of sulfation of HS is specific to the tissue and the moment of expression, and may be dynamically remodeled. For instance, high levels of 3-O-sulfated glucosamine were found in HS from follicular fluid, where nearly 50% of total HS is endowed with anticoagulant activity [2] and participates in the anticoagulant state of preovulatory follicular fluid. HS chains are usually attached to a core protein to form proteoglycans (HSPGs) that can be expressed on the cell membrane (syndecans and glypicans), or released in the extracellular matrix (ECM) where they constitute major structural molecules (agrin, perlecan, and type XVIII collagen) or be present in secretory vesicles [3]. 

HS is expressed on the cell surfaces of all mammalian cells where HSPGs participate in a variety of cellular processes and their functions depend on both HS and the protein core; for example, they serve as endocytic receptors and storage for macromolecules, such as lipid, growth factors, and morphogens [3,4], as modulators of cell mobility [5], and can participate in transportation of solutes between vessels and ECM (for review, see [6]). Also, heparan sulfate accounts for 60–90% of the total amount of the glycocalyx synthesized by endothelial cells [7] and whose anticoagulant and overall charge are very important for the repulsion of negatively charged molecules, including albumin, leukocytes, red blood cells, and platelets. Thus, the network of interactions of HS regulates fundamental biochemical and developmental processes and has been called the “HS-interactome” [8]. The last step of HS biosynthesis which is 6-O-desulfation by endosulfatases (SULF1 and SULF2), takes place on the cell surface and results in release of growth factors and cytokines immobilized on HSPGs and regulation of the effects of signal transduction [9,10]. In fact, the removal of sulfate groups from HS causes the release of HSPG-sequestered ligands that can act back on the cells and, indeed, overexpression of SULFs has been reported in a wide range of human tumors [11,12]. 

HSPGs expressed on the cell surface can be subjected to limited proteolysis and release in the medium or can be internalized by endocytosis and degraded by a multi-step process terminating in the lysosomes. The best characterized degradation pathway is that of ovarian granulosa cells in which two pathways have been described [13]. HSPGs anchored to the membrane by glycosylphosphatidylinositol (GPI) are quickly transported to lysosomes after endocytosis. Instead, HSPGs with transmembrane protein domains are first treated in prelysosomal compartments for removal of the protein core and partial degradation of HS chains. Secondly, HS chains are completely degraded in lysosomes. Differently, HSPGs in cultured rat hepatocytes have been shown to be long recycled back to the plasma membrane or to be associated with intracellular compartments before being degraded in lysosomes with no preliminary processing in early endosomes [14]. At the moment it is not clear if differences in HSPGs endocytosis reported in literature could be justified by the cellular context and the type of extracellular ligand.

**Figure 1:**
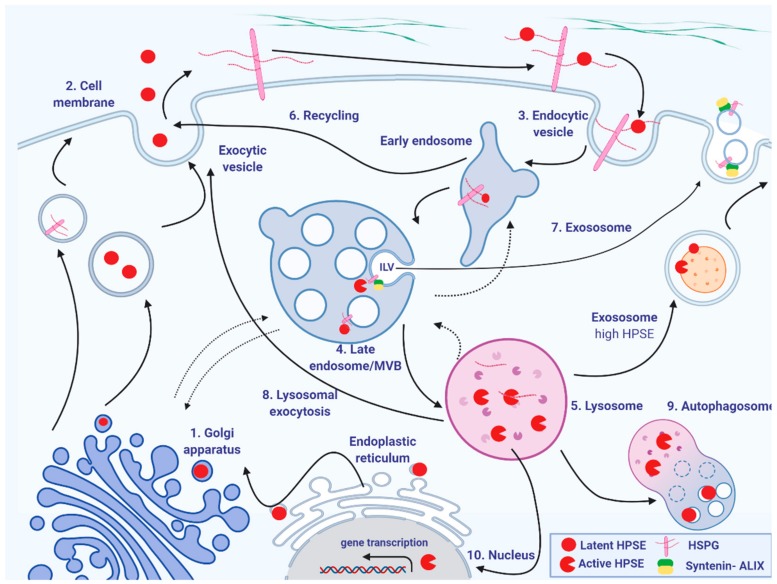
Schematic model of heparan sulfate proteoglycans and heparanase trafficking. (1) In the Golgi apparatus HS chains are polymerized and preHPSE is processed to produce proHPSE by the elimination of the N-terminal signal peptide. (2) The newly biosynthesized HSPGs are then shifted to the cell membrane where they can interact with the proHPSE and (3) the complex is rapidly internalized by endocytosis and then (4) accumulated in the late endosome. (5) Upon fusion of the late endosome with lysosome, proHPSE is activated and cleaves HS chains that are completely degraded by lysosomal hydrolases. (6) HPSE and HSPGs can be recycled to the cell surface from endosomes. It appears that active HPSE pursues other paths in the cells. (7) Trimming of HS from syndecans by active HPSE present in late endosomes leads to formation of the syndecan-syntenin-ALIX complex [50]. Intraluminal vesicles (ILV) are then formed by the invagination of endosomal membranes, resulting in the formation of multivesicular bodies (MVBs). MVBs release ILVs as exosomes upon fusion with the cell membrane and deliver their cargo to recipient cells. In the presence of high levels of HPSE, the enzyme can be found on the surface of exosomes and modulates tumor microenvironment. (8) Lysosomal exocytosis has been observed in malignant cells. (9) HPSE also regulates autophagy by driving fusion of lysosomes with autophagosomes which degrade macromolecules into monomeric units. (10) Perinuclear lysosomal HPSE can also translocate into the nucleus and regulate gene transcription and cell differentiation.

The only known mammalian endoglycosidase able to cleave HS is heparanase (HPSE), which is secreted as a latent 65-kDa enzyme, which rapidly binds to HSPGs and the complex is then internalized by endocytosis [15]. Heparanase uptake is a prerequisite for the delivery to lysosomes where it is activated by cathepsin L and cuts HS at a limited number of sites, specifically the β-(1,4) glycosidic linkage between GlcA and GlcNS, producing fragments that are 10–20 saccharide units in length and still able to interact with protein ligands [16,17]. Also, a second HPSE protein named heparanase-2, without enzymatic activity, has been discovered and can compete for the substrate thus inhibiting HPSE activity but, its role in healthy tissues is broadly unknown [18]. Upon heparanase action, another nine exo-enzymes are responsible for the rapid and complete degradation of HS fragments: five sulfatases, three glycosidases, and an integral membrane enzyme required for the transfer of acetyl groups [19]. The process is influenced by the structure of the to-be-degraded HS: in CHO mutant cells, HS bearing reduced 2-O-sulfation was less cleaved by intracellular heparanase thus allowing recycling of unprocessed proteoglycans. In fact, higher amounts of extracellular HS chains were detected and accounted for the missing intermediate and small fragments observed inside the cells [20]. It has also been shown that heparanase drives fusion of lysosomes with autophagosomes thus controlling the basal levels of autophagy and contributing to maintain homeostasis [21] (Figure 1). Although numerous studies have accumulated regarding HS turnover especially in the last years, there is still debate on certain aspects which remain unclear. For example, the exact location and moment of activation of heparanase, whether in late endosomes or lysosomes, is still not precisely defined, together with the mechanism of HSPGs trafficking. We can speculate that a certain number of possibilities are available rather than a single linear mechanism, all converging to a final aim which is the reaction of the cell to different types of stimuli. 

Synthesis and degradation of HS are both complex pathways involving a variety of different enzymes, spatially and temporarily tuned to produce heterogeneous chains with different sulfation degree but tissue-specific expression of ligand-binding sequences. The overall pattern of HS modification appears relatively constant within a specific cell type or organ; therefore, the control of HS biosynthesis must be strictly regulated [22]. Not unexpectedly, altered levels of HSPGs or altered expression of genes coding for HS biosynthetic enzymes have been associated with the development of many diseases [23,24,25,26,27,28,29]. 

Today, all genes responsible for HS biosynthesis have been cloned and identified, which helps investigate the possible association of these genes with several pathologies. Also, mouse models in which different genes important for HS biosynthesis have been knocked out have helped understanding that genetic ablation of biosynthetic enzymes does not strictly correlate with a predicted phenotype in vivo [30,31], probably due to the existence of compensation mechanisms and to functional redundancy between gene family members involved in HSPG biosynthesis and redundancy in HS sequence–activity relationships. For example, ablation of the gene EXT1 in mice, which codes for a polymerase involved in the elongation of HS chains, resulted in loss of HS, disruption of gastrulation, and embryonic lethality before E8.5 [32]. In other cases, the outcome of a gene knockout may be complex as several enzymes occur in isoforms [22]. Mice that lack 3-O-sulfotransferase-1 (3-OST-1) do not show a procoagulant phenotype, although the 3-O-sulfate group is essential for the interaction of vascular HS with antithrombin [26]. Nevertheless, the other existing 3-OST isoforms have distinct substrate preferences, and may therefore regulate different biologic properties of HS [33,34]; therefore, the final phenotype is not explained by their compensatory activity. Actually, all experimental data accumulated cannot be summarized by a simple scheme.

### 1.2. The Use of HS Mimetics

Due to the pleiotropic effects that HS can exert and to the fact that HSPGs are ubiquitous, the idea of interfering with HS turnover can either be seen as the intriguing possibility to obtain multiple effects or the limiting fear that non-specificity would impact undesired pathways. According to most of the observations reported to date, only certain interactions rely on specific oligosaccharide sequences [35,36], whereas many others rely on a certain type of sulfation or negative charge density [37,38]. Nevertheless, the interest in HS-protein interactions is continuously growing also thanks to advance in analytical techniques and chemical synthesis of oligosaccharides with precise structures which bring light on the structural requirements for HS activity [39,40].

It is well known that heparin, which can be considered a highly sulfated version of heparan sulfate, has been used clinically for more than half a century as anticoagulant drug and has been shown to possess a number of beneficial effects in other diseases than thrombosis [41]. Prolonged administration of heparin can cause adverse effects, mostly related to bleeding [42], but also an immunological reaction, heparin-induced thrombocytopenia [43,44,45]. Other side effects are clinically irrelevant, despite the possible interactions that can be established following heparin diffusion into tissues, suggesting that they may be absorbed by the network of proteins with which heparin and heparan sulfate interact. These considerations support investigation on the use of heparin as a starting material to generate derivatives or HS mimetics as therapeutic agents to interfere with pathways in which HS is involved as a means of intervening in biochemical processes for medical purposes.

A plethora of different compounds both of GAG and not-GAG nature have been synthesized, the potential use of which has been comprehensively and recently reviewed [46,47,48]. A body of evidence indicates that the altered expression or activity of heparanase determines a profound impact on tumor behavior; indeed, heparin analogs able to inhibit heparanase have been developed to primarily target cancer and some proved very active and have been tested in clinical trials [49]. 

This review will focus on the possibility to control HS turnover by acting upon heparanase, at both extracellular and intracellular levels, a strategy that can be applied to many conditions, from inflammation to cancer and neurodegeneration. Many papers on HPSE have been written, and it is not our purpose to review exhaustively the biological literature on heparanase and the use of heparin derivatives, which has been undertaken successfully elsewhere [46,47,49].

Heparanase has a role intracellularly where it is involved in the catabolism of HS but it is also present extracellularly. On one hand, heparanase is overexpressed in many tumors and the active form of the enzyme is released extracellularly, therefore, explaining the rational of employing HPSE inhibitors to block tumor progression. On the other hand, in different conditions such as mucopolysaccharidosis, and, in particular, Sanfilippo syndrome, which is a neurodegenerative lysosomal storage disease, the use of HPSE inhibitors may impact HPSE action and influence the global distribution of HSPGs.

## 2. HS in Neurodegenerative Disorders

### 2.1. Different Involvement of HS in Neurodegenerative Disorders

Convergent phenotypes have been observed among the major pathologies affecting the brain. Neurodegenerative diseases are characterized by progressive accumulation of specific protein aggregates in the brain, e.g., amyloid-β (Aβ) in Alzheimer’s disease (AD), α-synuclein in Parkinson’s disease (PD) [51], and proteins with expanded polyglutamine (polyQ) in Huntington’s disease (HD) [52]. According to the concept of “seeding”, soluble proteins with nonnative conformations could assemble to form long unbranched structures called amyloid fibrils, able to trigger further incorporation of monomers into the fibrils. Regardless of the nature of the amyloid protein, HS has been found in all extracellular amyloid deposits [53,54], and HSPGs have been shown to be present at all stages of the degeneration process: neuron, intracellular and extracellular neurofibrillary tangles (NFTs). These fibrillary aggregates are formed by hyperphosphorylated forms of the microtubule-associated protein tau and have been detected in many neurodegenerative disorders, including AD [53,55,56,57,58,59]. Membrane-associated HS participates in the uptake and internalization of tau seeds [54], and concomitant hyperphosphorylation of tau was observed in neuroblastoma cultures [60], justifying the possible association with tau neurons. Moreover, cell surface HS directly binds fibrillar Aβ [61] and mediates its stabilization, internalization and deposition [62,63]. Evidence for HS participation in the development of amyloid disorders was also confirmed by overexpression of heparanase which resulted in inhibition of HS–amyloid interactions [64,65]. Accumulation of HS was also observed in neuronal nuclei in AD brain and it ultimately ended in altered protein expression and neuronal dysfunction [66]. Interestingly, HS may play distinct and partly contradictory roles in Aβ aggregation and deposit. In fact, binding of HS to the enzyme β-secretase (BACE1), involved in the liberation of Aβ peptide from the amyloid precursor protein (APP), prevents proteolytic cleavage and therefore the formation of Aβ peptide [67]. A comprehensive review has been recently written on HS and tauopathies [68].

HS mediates the fibril uptake of tau and α -synuclein but not Hungtington fibrils, suggesting that the mechanism depends on cell type and on certain type of sulfation of HS but not on specific sequences [69]. Castillo et al. showed that removal of O-sulfates led to a significant loss of heparin-enhanced Aβ fibrillization [70], whereas later Zhang et al. observed the presence of 6-O-sulfated glucosamine residues within the HS sequence interacting with Aβ [71], and recently 6-O sulfation was identified as a major determinant of tau binding in vitro [72]. Finally, the central role of 3-O-sulfation in tau abnormal phosphorylation was recently discovered by inhibiting the HS3ST2 enzyme in zebrafish, which resulted in arrest of tauopathy and animal functional recovery [60]. 

APP and Aβ are degraded (or recycled) in part by an endosomal–lysosomal pathway [73,74,75], and lysosomes are responsible for degradation of internalized aggregates in both neurons and in glial cells [76]. Efficient clearance of extracellular protein aggregates would prevent their transfer between cells, whereas compromised lysosomal efficiency may impair the degradation of many molecules normally processed within these organelles. Indeed, deficiency of lysosomal hydrolases is associated with the development of many human diseases, referred to as lysosomal storage disorders, which are often characterized by neurodegeneration [77]. Particularly, shortage or lack of specific lysosomal GAG-degrading enzymes impairs the autophagosome–lysosome fusion and leads to accumulation of GAGs; these diseases are called mucopolysaccharidoses (MPS) and many subtypes have been identified depending on the type of GAG and the specific enzyme affected. MPS III, called also Sanfilippo syndrome, is caused by deficiency of the lysosomal enzyme N-sulfoglucosamine sulfohydrolase (SGSH), resulting in incomplete degradation of HS and dramatic effects on the central nervous system with devastating consequences. Undegraded HS can affect cell functions not only due to its storage into lysosomes, but also when present outside the cell; probably, failure to degrade GAGs in MPS results in secretion and deposition in the ECM, as dysregulation of the ECM has been identified in several case reports [78,79]. Increased levels of protein markers associated with AD and PD have been detected in the brains of MPSIII mice: deposition of hyperphosphorylated tau and tau kinase, lysozyme, amyloid-β and amyloid precursor protein are all evident [80]. Moreover, α-synuclein aggregation and accumulation with neurons were observed both in MPSIII patients [81,82] and in MPSIIIA mice [83,84]. 

### 2.2. The Use of HS Derivatives in Neurodegenerative Disorders

Therapeutic approaches that target the central nervous system (CNS) are challenging due to the presence of the blood–brain barrier (BBB), which in healthy brain impedes most compounds from transiting from the blood to the brain. BBB permeability is influenced by molecular weight, charge, lipid solubility, surface activity, and relative size of the molecule [85]. If active compounds are small enough or lipid soluble they can pass the BBB by passive diffusion, alternatively they may be actively uptaken by receptors, transporters or carriers expressed by the cells of the BBB (endothelial cells, pericytes, astrocytes and neurons). The components of the BBB continuously adapt in response to various physiological changes in the brain and the integrity of the BBB is compromised in many pathological conditions including PD, AD, and MPS [81,86,87]. As a consequence, cytokines and immune cells could enter the CNS, activate glial cells, and cause alterations in the extracellular environment with consequent inflammation and damage [88,89]. 

A body of evidence supports targeting of HS-protein interactions in tauopathies as a therapeutic strategy. As already mentioned, studies on amyloidosis showed that overexpression of heparanase resulted in disruption of the HS–amyloid interactions, suggesting a potential strategy to interfere with the formation of fibrils. Indeed, administration of exogenous GAGs could competitively inhibit the harmful processes mediated by endogenous GAGs [90] and, in fact, heparin disaccharides were shown in vitro to pass the BBB and to efficiently decrease Aβ deposition [91], whereas chemically synthesized heparin-like oligosaccharides up to decasaccharides proved able to reduce the uptake of tau oligomers limiting their infectivity [92]. Neuroparin, a mixture of oligosaccharides with average MW of 2.1 kDa obtained by gamma irradiation of heparin, crosses the blood–brain barrier and was reported to attenuate the abnormal tau immunoreactivity in rat hippocampus and to have neuroprotective effects in several animal models of AD [93,94]. These compounds were small enough to cross the BBB and indeed an important consideration for therapeutic purposes is the extent of bioavailability of the administered GAG in the CNS. Nevertheless, in inflammatory conditions and highly damaged tissues, it is probable that increased permeability of the BBB would allow the entrance of compounds that would normally be rejected. 

### 2.3. The Possible Use of HS Derivatives in MPS III

In MPS III disease, neurodegeneration is a consequence of HS accumulation in lysosomes [95]. To date, no treatments have shown promise in modifying the progression of this exceptionally rare, but ultimately lethal, disease. A review has been recently published summarizing treatment approaches for the brain in MPS [96]; therefore, only a brief summary on possible therapies will be treated in this paragraph, with a final focus on the use of heparin derivatives.

The promising Enzyme Replacement Therapy (ERT), which works for MPS I, MPS II, MPS IVA, and MPS VI, is more difficult in MPS III, where it should cross the blood–brain barrier. The approach is based on systemic delivery of recombinant lysosomal hydrolases which are internalized through mannose-6-phosphate receptors [97,98,99]. Intravenously delivered enzyme has been shown not to cross the BBB in an adequate amount to prevent progression of neurological manifestations [100]. Gene therapy as a treatment for MPS can potentially provide a stable and continuous source of enzyme in combination with immunosuppression to prevent an immune response against the vector. Indeed, intracerebral injection of the vector AAVrh10-hMPS3A in four children with MPS IIIA and of the vector ABO-102 in six MPS IIIA patients showed good tolerability and evidence of decreased neurocognitive decline [101]. Fusion of the recombinant enzyme to a monoclonal antibody against the human insulin receptor has been shown by Boado and coworkers to facilitate the uptake in the brain by exploiting the transport mechanisms already present in the BBB [102,103]. Another strategy called substrate reduction therapy (SRT), aims to inhibit the early stage of the lysosomal degradation pathway, reducing GAGs synthesis [104]; however, results of a 6-month clinical trial with miglustat—an imminosugar used in the treatment of type I Gaucher disease—showed no improvement of cognition or behavior in MPS III patients [105]. Another approach under investigation is chaperone therapy, with the aim of partially restore the missing enzymatic activity. In fact, 10% activity is considered sufficient to prevent lysosomal GAG storage in MPS patients [106]. Lastly, also hematopoietic stem cells transplantation (HSCT) which consist in delivery of donor stem cells producing the deficient lysosomal enzyme, has been extensively studied over the past decades with variable results [107]. 

HS in MPS patients is not only accumulated in lysosomes, but seems to be redistributed to different cellular and extracellular localizations [108,109]. De Pasquale et al. recently published an innovative approach called substrate-masking technology based on sequestering of extracellular excess HS (and/or DS) with consequent restoring of the natural equilibrium. Treatment of primary fibroblasts from MPS IIIA- and MPS IIIB-patients with the protein NK1 resulted in decreased GAGs accumulation and restoration of FGF2 signaling [110].

As already suggested [111], targeting HPSE in lysosomes may be an interesting way to affect autophagy either by employing HPSE inhibitors, whose ability to cross the BBB is nevertheless still unclear, or by decreasing the lysosomal content of HPSE. For example, treatment with the inhibitor PG545 was shown to decrease autophagy and promote accumulation of HPSE extracellularly while reducing its level in the lysosomes [112]. Our strategy is to employ HPSE inhibitors as a mean to affect HS turnover, based on the idea that uncleaved HS chains may fuel recycling of HSPGs and lead to a lower amount of HS degraded in lysosomes. We have tested several HPSE inhibitors on primary fibroblast from KO MPS-IIIA and spontaneously mutated MPS-IIIA mouse models [113]. Indeed, treatment with these compounds could affect HS turnover, leading to decreased HSPGs production.

## 3. HS in Cancer

### 3.1. Expression of HSPGs in Tumorigenesis

Heparan sulfate and HSPGs are expressed on all eukaryotic cells, including cancerous cells and stromal cells surrounding the tumor and play an important role in tumor–stroma cross-talk, and thus in cancer development, transformation, growth, and metastasis. Therefore, alteration of the normal expression levels of HSPGs has often been described in tumors. Several HSPGs have been shown to be upregulated in many cancers [114]; for example, increasing expression level of Agrin in hepatocellular carcinoma [115] and of glypican-1 in pancreas carcinoma was detected and associated with poor prognosis [116]. Levels of glypican-1 and syndecan-2 are also increased in colorectal cancer [117]. Syndecan-1 has been demonstrated to regulate αvβ3 and αvβ5 integrin activation during angiogenesis and tumorigenesis on mammary carcinoma cells [118], whereas shedding of syndecan-2 has an antiangiogenic effect on the endothelium [119]. Moreover, breast cancer was found to upregulate glypican-1 [120] and syndecan-4 [121] and to downregulate glypican-3 [122], whereas low expression of glypican-3 promotes tumor proliferation and metastasis [123]. Contrarily, glypican-3 was found to be overexpressed in approximately 70% to 80% of hepatocellular carcinomas [124] and high glypican-5 expression levels in non-small cell lung cancer were associated with poor differentiation, vascular invasion, regional lymph node metastasis, and a higher TNM stage (TNM classification of malignant tumors) [125]. In addition, glypican-2 is upregulated in neuroblastoma and associated with poor overall survival [117].

In general, remodeling of HSPGs through enzymatic modification of HS chains is associated with malignant transformation of cells and can potentially serve as molecular biomarker to aid in the diagnosis and prognosis of cancer [114,126]. Also, alteration of glycosylation affects cellular adhesion and is associated with oncogenic transformation and metastasis [114]. Indeed, altered HSPGs glycosylation has been described in lung and brain cancer [127].

### 3.2. Tumor–Stroma Cross-talk

Tumor stroma is composed of the ECM including proteoglycans, fibronectin, collagen, cytokines, and growth factors. As the tumor evolves, the stroma undergoes tissue remodeling under the action of enzymes able to modify the glycosidic chains of the ECM, i.e., glycosyltransferases, sulfotransferases, sulfatases, and heparanase [128]. The presence and amount of these GAG-related enzymes help to identify high-risk patients and to develop personalized therapeutics [129]. Indeed, Barkeer and collaborators demonstrated that elevated O-glycosyltransferases GALNT3 and B3GNT3 expression regulates cancer stem cell markers in pancreatic cancer and their knockdown leads to decreased clonogenicity and migratory capacity [130]. Increased heparanase expression is also often described to promote an aggressive tumor behavior via multiple mechanisms [71,83]. It has been demonstrated that tumors are more aggressive when developed in transgenic mice overexpressing heparanase (Hpa-Tg and MMTV-heparanase), whereas smaller tumors develop in Hpa-KO mice [95,120].

HPSE functions inside the cell to promote autophagy and tumor growth by driving fusion of lysosomes with autophagosomes thus controlling the basal levels of autophagy [111]. Heparanase is an important player not only in maintaining homeostasis, as a postsynthetic modification enzyme for HS structure but also for its non-enzymatic and HS-independent effects which may contribute to tumor aggressiveness [131,132]. Therefore, heparanase orchestrates cellular responses in both normal and pathologic conditions. HSPGs bind to numerous bioactive molecules, i.e., growth factors, cytokines, chemokines, enzymes, which are stored in the ECM and can be released upon cleavage by extracellular heparanase. This strategy is adopted by tumor cells to ensure rapid tissue response as a fast-acting mechanism independent from *de novo* protein synthesis that meets their needs to promote tumor growth, angiogenesis, peripheral immune tolerance and formation of a metastatic niche. Briefly, tumor cells can change the nature of the microenvironment and vice versa the microenvironment can affect how a tumor grows and spreads [133]. 

Heparanase promotes signal transduction, including Akt, STAT, Src, Erk, HGF-, IGF- and EGF-receptor signaling [131]. Moreover, HPSE regulates the transcription of many other factors spanning from proangiogenic (i.e., VEGF-A, VEGF-C, COX-2, and MMP-9), to pro-thrombotic (i.e., tissue factor), proinflammatory (i.e., TNFα, IL-1, and IL-6), profibrotic (i.e., TGFβ), mitogenic (i.e., HGF), and osteolyic (RANKL) [133,134,135]. In summary, more and more functions of HPSE are being discovered, thus confirming its importance also in normal cell processes and the need of controlling its action and expression. The presence of heparanase was reported in Langerhans cells [136], where its function still has to be elucidated, and in astrocytes in mice after ischemia, where it can participate in the repair process [137]. Interestingly, increased expression of heparanase was found in placentas with preeclampsia [138,139], where it would enhance the increase of VEGF release and it would influence the invasion of trophoblast, similarly to the invasion of cancer cells [140]. 

While heparanase upregulation by tumor cells is well documented, not enough attention has been given to the protumorigenic function of heparanase expressed by non-tumor cells residing in the tumor microenvironment. In fact, heparanase released from platelets, neutrophils and mast cells upon degranulation participates in ECM degradation, facilitating diapedesis and extravasation of inflammatory cells [141,142,143,144,145]. HPSE release can therefore be a strategy used by metastatic tumor cells to invade blood and lymphatic vessels. Moreover, HPSE was discovered to mediate TLR activation at the cell membrane, followed by Erk/p38/JNK activation therefore regulating cytokine expression by macrophages, their activation and function in tumorigenesis and cross-talk with the tumor microenvironment [146].

Tumor cells are able to influence the responses of surrounding healthy cells as demonstrated by experiments in which healthy lymphocytes were co-cultured with sera from breast cancer patients or media from MCF-7 cells. Increased expression of HPSE and secretion of exosomes was indeed observed, thus revealing the importance of cross-talk [147,148]. Exosomes serve as mediators for intercellular communication through the delivery of proteins, factors and HS chains, important for signaling processes. Heparanase overexpression dramatically increases exosome secretion in human cancer cells of myeloma, lymphoblastoid, and breast cancer [149]. 

It has been recently discovered that chemotherapy upregulates heparanase expression in myeloma surviving cells and induces secretion of chemoexosomes with heparanase loaded on surface [150]. These tumor chemoexosomes can remodel extracellular matrix by degrading ECM heparan sulfate and/or by transferring their heparanase cargo to cells where HS degradation will induce signal activation [150], resulting in enhanced secretion of an important myeloma growth factor, TNF-α, by macrophages. Additionally, heparanase stimulates the expression of MMP-9 via ERK signaling, promoting shedding of syndecan-1 proteoglycan (CD138) from the myeloma cell surface [150]. Shed syndecan-1 ectodomain was shown to capture VEGF and form a complex that activates integrin and VEGF receptors on adjacent endothelial cells thereby stimulating tumor angiogenesis [151].

### 3.3. Heparanase Targeting by Heparin and Its Derivatives in Cancer Therapy

As venous thromboembolism is a well-known cause of death in patients with cancer [152], heparin has been frequently used in the treatment of cancer-associated thromboembolism. Accordingly, accumulation of clinical evidence shows that cancer patients treated with unfractionated and low-molecular weight heparin (LMWH) survive longer than patients treated with other anticoagulants, especially patients in the early stage of the disease [153,154,155,156,157]. Heparin has been showned to possess anticancer, antiangiogenic, and antimetastatic activity [158,159], including inhibition of heparanase, blocking of P- and L-selectin-mediated cell adhesion, and inhibition of angiogenesis, but its anticoagulant activity and the possible side effects as bleeding and heparin-induced thrombocytopenia limit long-term treatment. As already mentioned, heparin derivatives or HS mimetics have been synthesized with reduced or absent anticoagulant activity but maintaining their binding selectivity potential towards a vast array of HS-binding proteins, many of which with pivotal roles in cancer growth and progression [46].

Nowadays the interest of researchers in oncology is not only limited to tumor cells but especially on tumor microenvironment. Information gathered on the two topics is so vast that they appear as two different specialized areas of research that are, on the contrary, profoundly interconnected. The separation into the following sections is based on experimental observations either directed on the tumor itself or on the components of the tumor microenvironment.

#### 3.3.1. Targeting the Tumor

Numerous clinical association studies have consistently demonstrated that upregulation of heparanase expression correlates with increased tumor size, tumor angiogenesis, enhanced metastasis, and poor prognosis [160,161]. In contrast, knockdown of heparanase or treatments of tumor-bearing mice with heparanase-inhibiting compounds markedly attenuate tumor progression [162,163,164], further underscoring the potential of anti-heparanase therapy for multiple types of cancer. 

Extensive research was done on targeting HS-degrading activity of heparanase [49,128,165,166]. The heparanase inhibitory effects of non-anticoagulant heparin have been described by Bar-Ner et al., suggesting that heparanase inhibition was dependent on polysaccharide size, degree and pattern of sulfation, and N-substitution of hexosamines [167]. 

Some supersulfated low-molecular weight heparins (ssLMWH) have been synthesized with low anticoagulant activity despite the high degree of sulfation and were shown to reduce synovial sarcoma growth and metastases in vitro and in vivo by interfering with the activity of heparanase, growth factor/receptor axes and proinflammatory molecules (e.g., leucocyte elastase and cathepsin G) [168]. Another compound, SSLMW-19, a key factor in the regulation of iron metabolism, and also involved in carcinogenesis and metastasis, was shown to rapidly and strongly inhibit the expression of hepcidin in vitro and in vivo [169].

Glycol-splitting of unsubstituted uronic acids obtained by periodate oxidation and borohydride reduction has been applied to a variety of heparin derivatives and increased the flexibility of the molecules for a better interaction and inhibition of heparanase or growth factors. M402 (Necuparanib) is a glycol-split LMWH with reduced anticoagulant activity [170] that was found to reduce tumor burden in vivo in KPFMC pancreatic cancer mice model at a dose of 40 mg/kg/day and reduce AsPC-1 pancreatic cancer cell line proliferation and invasion in vitro in a 3D-culture model [171]. Boothello et al. synthesized a non-saccharide compound, G2.2, which is structurally homogeneous and easy to obtain by chemical synthesis, as a mimetic of an HS hexasaccharide active on cancer stem cells. This HS mimetic reduced in a dose-dependent manner the growth of colon subcutaneous xenografts in mice and delayed the growth when added to oxaliplatin and 5-fluorouracil, which are the most frequently used chemotherapy treatment for colon cancer [172]. Also, polysaccharides from different sources are potentially able to interfere with tumorigenesis. For example, an oversulfated heparin-like polysaccharide from marine origin was recently evaluated for its ability to reduce cancer cell characteristics on human endometrioid cancer cell line in vitro. This compound effectively reduces the ability of tumor cells to initiate migration by inducing 51.6% of slowdown and induces 87% of inhibition of tumor cell invasion on Transwell compared to non-treated cells (unpublished data). It also inhibits endometrial tumor cell proliferation and is well tolerated at concentrations of 100, 75, 50, and 25µg/mL (unpublished data). A sulfated oligosaccharide mimetic of heparan sulfate, PI-88, was used to inhibit HS effector functions and heparanase activity. PI-88 reduced tumor volume by 66–70% in RIP1-Tag2 mice model of pancreatic islet cell carcinogenesis [173]. Joyce and collaborators also noticed decreased tumor cell proliferation, increased apoptosis and impaired angiogenesis associated with reduction of VEGF-A and its receptor VEGF-R2 on the tumor endothelium [173].

#### 3.3.2. Targeting the Tumor Microenvironment

Impact of heparanase on tumor progression is related to its function in mediating tumor-host cross-talk, priming the tumor microenvironment to better support tumor seeding and growth [174]. Targeting of heparanase in the tumor microenvironment is a promising strategy to restrain tumor growth and dissemination. Indeed, a study showed the effect of heparanase-neutralizing antibodies to attenuate the growth of lymphoma cells that do not express heparanase, implying that targeting microenvironment may be sufficient [175]. 

Heparin derivatives and HS mimetics can also affect invasion and metastatic tumor behavior by counteracting HSPGs functions.. Yoshitomi et al. observed reduced tumor growth dissemination on several murine metastatic models by employing a glycol-split weak anticoagulant heparin [176].

Another strategy to contain metastatic progression could be targeting the angiogenic pathway. A low-molecular-weight glycol-split heparin (ST2184), generated by nitrous acid depolymerization of an undersulfated glycol-split heparin derivative (ST1514), was shown to act as VEGF antagonist by exploiting binding to VEGF165 while preventing receptor engagement [177]. This molecule inhibited neovascularization in the chick embryo chorioallantoic membrane, metastatic dissemination to the lung in the B16-BL6 mouse model of melanoma and significantly reduced angiogenesis of human MeVo melanoma xenografts [46,177]. Naggi and coworkers developed a 100% N-acetylated and 25% glycol split heparin, known as SST0001 (Roneparstat) [178], which is a potent heparanase inhibitor that is also able to suppress angiogenesis and to downregulate HGF, VEGF, and the expression of ECM-remodeling proteins (Matrix metalloproteinase 9, pentraxin, and urokinase-type plasminogen activator) [179]. Also, in combination with dexamethasone it was able to inhibit myeloma tumor growth in vivo through dual targeting of the tumor and its microenvironment via the heparanase/syndecan-1 axis [180].

We previously mentioned that M402 heparin mimetic effectively targeted pancreatic tumor cells. Interestingly, MacDonald et al. observed that M402 also targeted the stromal compartment. On a co-culture of pancreatic tumor cells and stellate cells that mimics epithelial–stromal interactions, treatment of M402 significantly decreased the invasive behavior in a dose-dependent manner, whereas in vivo it extended survival and reduced metastasis by reducing the protein levels of matrix metalloprotease 1 and by increasing the tissue inhibitor of metalloproteinase 3 (TIMP3) [171]. Karoli et al. demonstrated that carbohydrate-based HS-mimicking compounds, PI-88 and analogues, inhibit heparanase and compete with growth factors (FGF-1, FGF-2, and VEGF) for binding to HS, and therefore impact anti-angiogenic and anti-metastatic activity [48,181]. Another octasaccharide-based heparin mimetic synthesized by Dollé and coworkers was characterized in vitro for its binding affinities and towards VEGF-A, FGF-2, PDGF-β, and SDF1-α using the BIAcore technology and for the potential inhibition in an heparanase activity assay [182]. This novel molecule could thus potentially inhibit angiogenesis by interfering with components of the extracellular matrix. Lim and collaborators developed saccharide-free polyproline-based GAG mimetics (PGMs) that recapitulate key GAG structural features, notably the sized repeating units, periodicity, and helicity. GAG activities were also maintained, indeed PGMs effectively inhibited chondroitin sulfate-E binding to P-selectin which is implicated in metastasis, thrombosis and inflammation and successfully attenuate hematogenous metastasis in vivo in mice model as effectively as heparin and tinzaparin without any adverse effects on mice model, suggesting its safe use in vivo [183].

### 3.4. Clinical Considerations

Over the last 30 years of active research, there has been an explosion of information about the molecular biology of cancer. The challenge remains to translate this information into advances in patient care to convert new molecular information into drug therapy. According to all these studies, high levels of heparanase could be used as predictive and prognostic molecular marker for cancer. In a study published in 2016, high level of HPSE in the melanoma metastases predicted poor prognosis in patients stage IVc [184]. However, HPSE expression in the primary tumor does not always reflect metastatic output. This is the case in breast cancer patients, where the primary lesion stained positive for heparanase in some cases while the metastasis stained negative, and vice versa [185]. In addition, we observed high HPSE expression in all primary lesions from endometrioid cancer grade 1 patients despite different patterns and degree of invasions. 

A number of preclinical and clinical studies have suggested that heparin and LMWH treatment in addition to conventional treatment significantly improves overall survival in cancer patients with advanced stage cancer but increasing the risk for bleeding complications [186,187]. Regarding the use of heparanase inhibitors to block tumor progression and their weak anticoagulant activity, Muparfostat PI-88 was the first heparanase inhibitor tested in early stage clinical trials in cancer patients in 2011. PI-88 was administered both alone and together with docetaxel to cancer patients in two Phase 1 studies validating their tolerance and safety for the patients [135,136]. PI-88 was also the first to enter clinical trials reaching phase III, where it was administered as an adjuvant therapy for hepatitis virus related hepatocellular carcinoma (HV-HCC) after its surgical resection. PI-88 prolonged the disease-free survival in the microvascular invasion subgroup (40% of the trial population) and exhibited anti-inflammatory properties [137]. Liao et al. demonstrated that heparanase was upregulated in both postsurgical plasma of HCC patients and in orthotopic mouse model induced after hepatectomy [137]. Upregulation of HPSE enhanced the sensitivity of HCC cells to PI-88 and thus the inhibitory effect of PI-88 on cell proliferation and migration. Likewise, Ramani et al. also showed that heparanase is upregulated in response to chemotherapy in myeloma patients and the surviving cells acquire drug resistance due to heparanase-mediated ERK signaling [138].

SST0001 heparin mimetic effectively inhibited myeloma growth in vivo, even when confronted with an aggressively growing tumor within human bone [180]. An open-label, multicenter, phase I, first-in-human study was designed to assess the safety and tolerability profile of Roneparstat in patients with relapsed/refractory multiple myeloma, and was well tolerated by patients exposed to the drug at dose levels of 200 and 400 mg/day without any clinically relevant toxicities [188]. Consequently, Roneparstat was found to be highly effective and overcome initial chemoresistance when used in combination with chemotherapeutic agents against established and aggressive myeloma tumors growing within bone or to treat brain metastatic breast cancer [189,190].

Analysis of plasma samples of patients with metastatic pancreatic cancer enrolled in a phase I/II study show that treatment with necuparanib in addition to the standard of care significantly increased TIMP3 plasma protein levels confirming the in vivo and in vitro studies on pancreatic cancer models [171]. 

PG545 has been selected over the PG500 series of heparan sulfate mimetics for the ability to inhibit both angiogenesis and heparanase activity and was selected as the lead clinical candidate for oncology [191]. Eight years after, PG545 was then evaluated on a Phase I study in patients with advanced solid malignancies that had relapsed or was refractory to standard therapy, and showed a disease control up to 24 weeks in 38% of evaluable subjects with increases in innate immune cell activation, plasma IFNγ, TNFα, IP-10, and MCP-1 [192]. 

These heparin mimetics that inhibit heparanase enzymatic activity are being evaluated in numerous clinical trials for various types of cancer, and appear to be well tolerated and also beneficial in combination with conventional anticancer drugs, thus providing a strong rationale for applying anti-heparanase therapy. Another strategy uses heparanase-neutralizing monoclonal antibodies to target the interaction of heparanase with HS and has been tested in pre-clinical studies for various types of cancer, including myeloma, pancreatic carcinoma, and hepatocellular carcinoma [175]. These heparanase-neutralizing mAbs profoundly attenuated myeloma and lymphoma tumor growth and dissemination of tumor xenografts produced by human lymphoma cells in preclinical models [175].

Although clinical trials focus on cancer patients, the same heparanase inhibitors are likely to be applied in other applications as chronic inflammation, sepsis, autoimmune diabetes, diabetic nephropathy, bone osteolysis, pancreatitis, viral infection, acute kidney injury, thrombosis, and atherosclerosis, as well as various rare diseases like mucopolysaccharidosis. Additional opportunities for development of anti-heparanase therapeutics include monoclonal antibodies and the use of the recently published crystal structure of heparanase for identification of small inhibitory molecules [81].

## 4. Conclusions

We gathered existing data around the idea of interfering with HS turnover by affecting heparanase and, indeed, the literature does support this working hypothesis. It is hoped that the review will promote discussion regarding the concrete possibility to therapeutically use HS mimetics/heparin derivatives which are able to act on existing pathways and to modulate them. We are confident that new discoveries will help clarify the aspects of HS turnover which are still foggy.
